# Kaposi varicelliform eruption: an unusual presentation caused by varicella zoster virus in a healthy adult patient - a case report

**DOI:** 10.1186/s12879-024-09115-4

**Published:** 2024-02-22

**Authors:** Hassan El-Masry, Safia Essam, Hamed Gaber, Nour Shaheen, Ahmed Abdelbary, Naglaa Mohamed Sayed, Salma Samir Omar

**Affiliations:** 1 Faculty of Medicine, Alexandria University, Alexandria, Egypt; 2 Department of Dermatology and Andrology, Faculty of Medicine, Alexandria University, Alexandria, Egypt

**Keywords:** Kaposi varicelliform eruptions, Eczema herpeticum, Viral skin infection, NSAIDs

## Abstract

**Background:**

Kaposi Varicelliform Eruptions (KVE), also known as eczema herpeticum, is a rare and potentially life-threatening dermatological condition primarily attributed to herpes simplex virus (HSV) infection, with less frequent involvement of Coxsackie A16, vaccinia, Varicella Zoster, and smallpox viruses. Typically associated with pre-existing skin diseases, especially atopic dermatitis, KVE predominantly affects children but can manifest in healthy adults. Characterized by painful clusters of vesicles and sores on the skin and mucous membranes, it often masquerades as other dermatological disorders. Non-steroidal anti-inflammatory drugs (NSAIDs) are commonly used for pain relief and inflammation, though their potential role as KVE triggers remains uncertain.

**Case report:**

Here, we present a case of an 18-year-old female with KVE attributed to Varicella Zoster virus (VZV) and successfully treated with oral acyclovir within a week, underscoring the significance of early recognition and intervention. KVE can manifest with systemic symptoms like fever, fatigue, and lymphadenopathy and may involve multiple organ systems, necessitating possible antibiotic use for complications.

**Conclusion:**

This case underscores the importance of prompt KVE identification and consideration of antiviral therapy to enhance patient outcomes. Further research is warranted to elucidate predisposing factors for this rare condition.

## Introduction

Kaposi Varicelliform Eruptions (KVE), also known as eczema herpeticum, is a rare and potentially severe cutaneous viral infection primarily attributed to herpes simplex virus (HSV1) or (HSV2) infection [1, 2]. The precise pathogenesis of KVE remains incompletely elucidated, yet it is recognized to be influenced by factors such as immunodeficiency and underlying dermatological conditions. Characterized by its distinctive cutaneous manifestations, KVE typically manifests in regions where the cutaneous barrier is compromised due to pre-existing dermatoses. While it predominantly afflicts pediatric populations, notably those with atopic dermatitis, it is not exclusive to this demographic, as cases have been documented in association with various underlying diseases, including ichthyosis vulgaris, pemphigus foliaceus, Grover’s disease, Darier’s disease, mycosis fungoides, Hailey-Hailey disease, and seborrheic dermatitis. Clinically, KVE presents as an abrupt eruption of painful clusters of vesicles and pustules across the skin, which can progress into crusted, hemorrhagic, and profound erosions. These lesions may coalesce to form extensive denuded areas, thereby heightening the risk of secondary bacterial infections. In addition to the local cutaneous manifestations, KVE can precipitate systemic symptoms, encompassing fatigue, elevated fever, and lymphadenopathy. Notably, KVE has the capacity to involve multiple organ systems, including the central nervous system, liver, lungs, gastrointestinal tract, and adrenal glands, potentially giving rise to diverse and intricate complications [3, 4].

In this report, we present the case of an otherwise healthy 18-year-old female patient, underscoring the imperative significance of a comprehensive exposure history. This case not only highlights the importance of considering less common viral etiologies for KVE but also underscores the potential absence of overt immunodeficiency or associated dermatological diseases. Moreover, we draw attention to the critical role of recognizing the distinctive clinicopathologic features characteristic of KVE. Additionally, we explore the intriguing possibility of NSAID use as a potential trigger in the context of diagnosing and managing Kaposi Varicelliform Eruption.

## Case presentation

### Patient information

The clinical presentation involved an 18-year-old female who developed a fever of 38 °C and an extensive skin rash over three days. She also displayed multiple superficial erosions in the oral cavity, impacting her ability to drink and swallow, and reported recent-onset low back pain. Notably, the patient had recently used non-steroidal anti-inflammatory drugs (NSAIDs) for pain relief before the onset of the rash. Importantly, there is no recorded medical history beyond NSAID usage. Including information about the patient’s medical history, underlying conditions, or family history of similar ailments could provide additional context for a more comprehensive evaluation of the case.

### Clinical examination

**Fig. 1:**
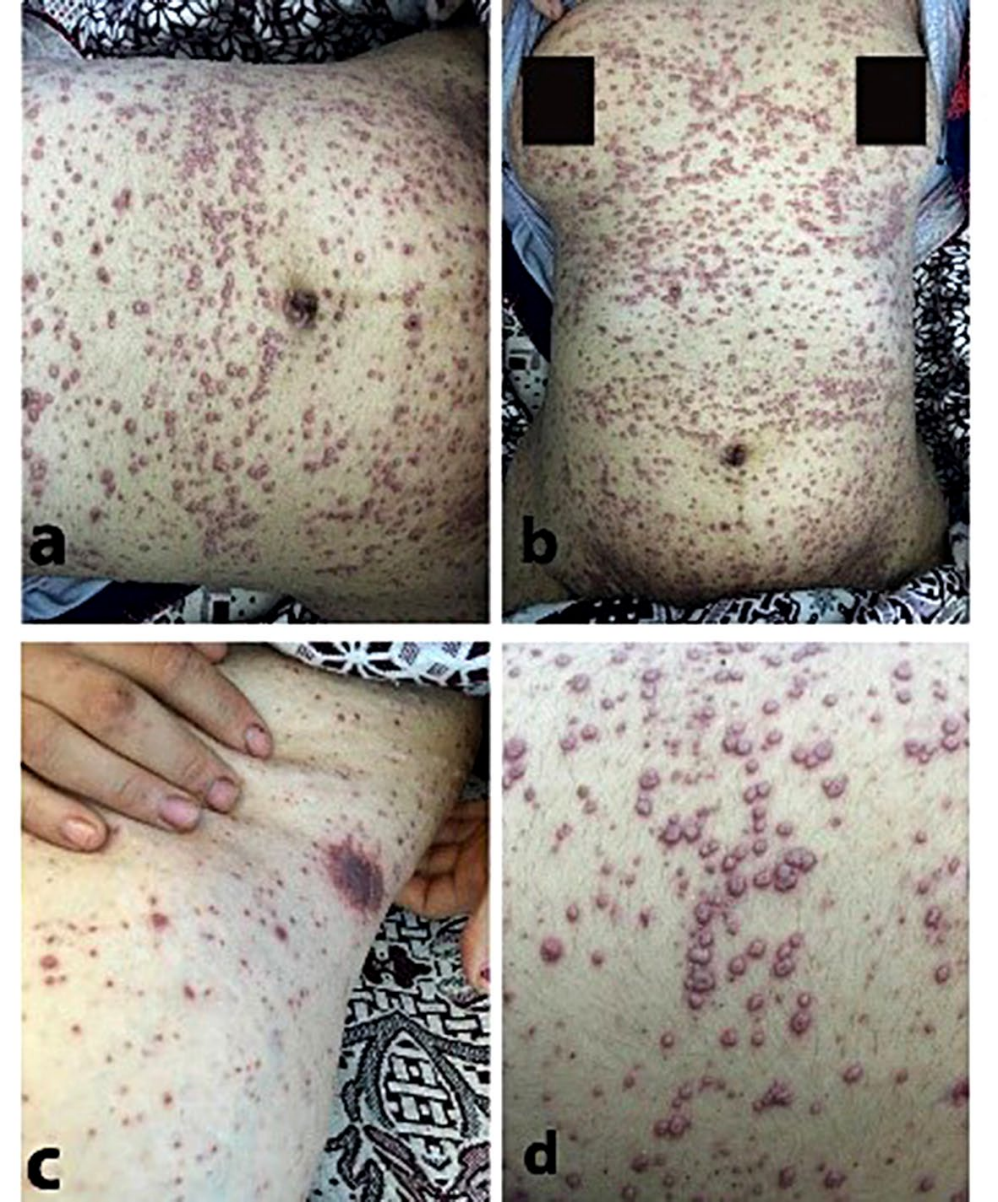
Monomorphic vesicular skin eruption with central umbilication (inside circle) over the abdomen (**a**, **b**), and the back (**d**), crusted erythematous plaque over chest (**c**)

In Fig. 1d, a detailed close-up photograph of the patient’s back vividly illustrates the monomorphic nature of the lesions, characterized by uniformly distributed discrete vesicles across the trunk, face, ears, and extremities. Notably, there is a distinctive absence of involvement on the palms and soles. Some vesicles exhibit central umbilication, particularly prominent on the trunk (Fig. 1), emphasizing the characteristic features of Varicella. Facial manifestations include significant eyelid swelling, larger vesicles, and bullae formation on the forehead. Coalescence of vesicles results in erythematous crusted patches, with a subset containing turbid fluids. Mucous membrane involvement extends to both the oral and genital regions. Importantly, there is no itching reported. This detailed image underscores the consistency and uniformity in the morphological presentation of the lesions, providing a precise representation of severe Varicella in this adolescent.

### Investigations and treatment

**Fig. 2:**
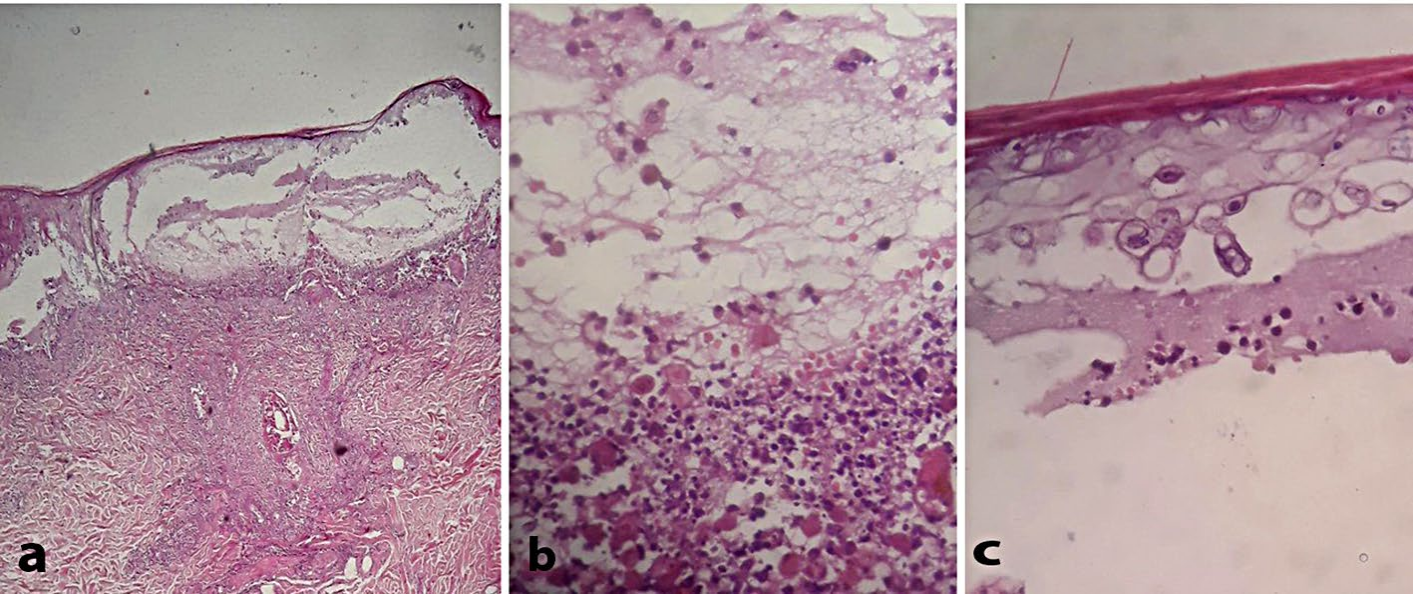
Multilocular Intraepidermal vesicles H&E 100 x (**a**), Viral cytopathic effect (reticular and ballooning degeneration) H&E 200x (**b**), Acantholytic ballooned cells with nuclear molding H&E 400x (**c**)

A complete blood count (CBC), as detailed in Table 1, showed an increased basophil count, there was no evidence of eosinophilia, with the eosinophil count measured at 0.024.” to highlighted 1st line in the Investigation and treatment section. Peripheral blood smear demonstrated no eosinophilia or atypical lymphocytes. Importantly, the absence of diagnostic criteria for drug reaction with eosinophilia and systemic symptoms (DRESS), including fever exceeding 38.5 °C, lymphadenopathy, eosinophilia, and atypical lymphocytes, ruled out this diagnosis [5]. Liver function tests revealed elevated levels of aspartate aminotransferase (AST) at 84 IU/L and alanine aminotransferase (ALT) at 97 IU/L, along with a mild increase in serum creatinine attributed to dehydration, which improved with intravenous fluid administration. A skin biopsy from the inner thigh yielded key histopathological findings, including vesicles in the upper layer of the skin with acantholytic cells, reticular degeneration of the epidermis, and ballooning degeneration of keratinocytes (Fig. 2). In conjunction with the clinical presentation, these findings led to the diagnosis of Kaposi Varicelliform Eruption. Further serological analyses revealed elevated levels of Varicella zoster immunoglobulin M (IgM), measuring 25 IU/ml via enzyme-linked immunosorbent assay (ELISA), surpassing the typical range of < 10 IU/ml [6]. Consequently, a comprehensive assessment incorporating clinical, histopathological, and serological factors confirmed the diagnosis of Kaposi Varicelliform Eruption secondary to Varicella Zoster Virus infection.

**Table 1:** Complete blood count (CBC)

Test description	Observed Value	Unit	Reference Range
Erythrocytes	4.64	10*6/ uL	3.8–4.8
Haemoglobin	12.8	g/dl	12–15
Haematocrit	38.7	%	36–46
M.C.V	83.5	fl.	83–101
M.C.H	27.6	Pg	27–32
M.C.H.C	33.1	g/dl	31.5–34.5
Leukocytes	12.14	10*3/ uL	4–10
Neutrophils	4.990	10*3/ uL	2–8
Eosinophils	0.024	10*3/ uL	0–0.6
Basophils	0.425	10*3/ uL	0–0.11
Lymphocytes	4.844	10*3/ uL	1.5–4
Monocytes	0.401	10*3/ uL	0.08–0.88
Platelets	107	10*3/ uL	150–410

M.C.V (mean corpuscular volume), M.C.H (mean corpuscular haemoglobin), M.C.H.C (Mean corpuscular hemoglobin concentration)

**Fig. 3:**
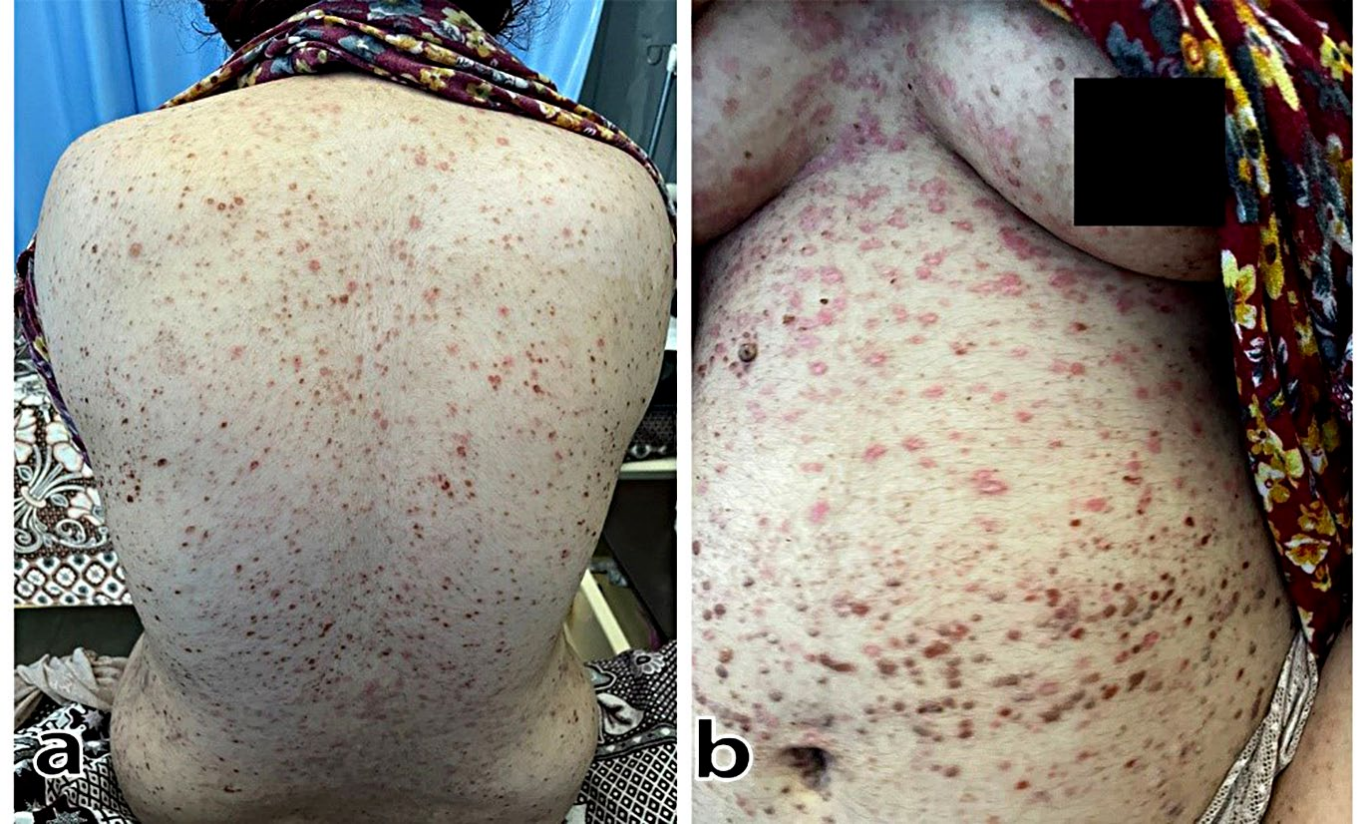
Patient follow-up after a week of antiviral treatment, improvement of the body popular skin eruptions (**a**, **b**)

Upon admission, the patient was promptly administered intravenous fluids to alleviate dehydration. Subsequent to the receipt of the skin biopsy results, treatment initiation commenced with oral acyclovir at a dosage of 800 mg, administered five times daily, beginning on the third day of admission [6]. Notably, a significant improvement in the patient’s condition, marked by the resolution of facial edema and blisters and substantial amelioration of skin eruptions, was observed by the seventh day of treatment. The patient expressed satisfaction with the progress, as visually depicted in Fig. 3.

## Discussion

The presented case underscores an unusual yet significant manifestation of Kaposi varicelliform eruption (KVE) in an otherwise healthy adult, attributed to Varicella Zoster Virus (VZV) infection following the intake of non-steroidal anti-inflammatory drugs (NSAIDs) [1, 2]. KVE is typically associated with viral infections, primarily herpes simplex virus (HSV1 or HSV2), but can also be triggered by other viruses, including VZV [1, 2]. The patient’s recent NSAID use initially raised suspicions of drug reaction with eosinophilia and systemic symptoms (DRESS) syndrome or acute generalized exanthematous pustulosis (AGEP) [5]. However, further investigations, including the absence of eosinophilia and atypical lymphocytes in DRESS and histopathologic findings devoid of intraepidermal, intracorneal, and subcorneal pustules in AGEP, ruled out these differential diagnoses.

The clinical presentation of the patient was characterized by a monomorphic vesicular eruption, often on an erythematous base, exhibiting central umbilication and pustulation in some lesions. These vesicles coalesced to form crusted erythematous patches distributed across the back, abdomen, extremities, and neck. Additionally, facial involvement was marked by eyelid edema and bullae formation. Mucous membrane lesions were evident in the oral and vaginal regions, along with bullae formation on the right inner thigh and neck. Importantly, the patient had no lymphadenopathy or prior history of herpes zoster infection.

The diagnosis of KVE attributed to VZV infection was established through a comprehensive analysis, including clinical evaluation, histopathological examination, and serological assessment. Classical histopathological findings [4], combined with the presence of VZV-specific immunoglobulin M (IgM) antibodies in the patient’s serum [7], confirmed the viral etiology of the eruption. Early initiation of antiviral therapy with acyclovir played a pivotal role in resolving facial edema, blister formation, and papular skin eruptions (Fig. 3), leading to substantial improvement within one week.

Notably, the potential role of NSAIDs in triggering KVE warrants further investigation. In vitro studies by Cheung et al. [8] and Giagoudakis et al. [9] have demonstrated a decline in neutrophil function following NSAID exposure. Whether this NSAID-induced compromise in antiviral defense mechanisms may exacerbate VZV infection and precipitate KVE in an otherwise healthy adult remains a subject of inquiry. A study by Mikaeloff et al. [10] has suggested an association between NSAID use and an increased risk of severe skin and soft tissue complications in varicella zoster virus (VZV) infection, particularly observed in children with varicella. However, it remains unclear whether NSAID ingestion by our patient led to a decline in neutrophil function, potentially contributing to the onset of KVE, despite her overall good health without underlying skin diseases.

## Conclusion

This case report sheds light on the clinical aspects, diagnostic considerations, potential triggers, and therapeutic interventions associated with Kaposi varicelliform eruption in the context of Varicella Zoster Virus infection. Emphasizing the relevance of considering KVE as a differential diagnosis in healthy non-immunocompromised adults presenting with generalized vesicular eruptions, it underscores the need for further research to elucidate the relationship between NSAIDs and KVE in VZV-infected individuals.

## Data Availability

All related data are available in the presented case.
